# Factors contributing to longer length of stay in Aboriginal and Torres Strait Islander children hospitalised for burn injury

**DOI:** 10.1186/s40621-020-00278-7

**Published:** 2020-10-05

**Authors:** Courtney Ryder, Tamara Mackean, Kate Hunter, Kurt Towers, Kris Rogers, Andrew J. A. Holland, Rebecca Ivers

**Affiliations:** 1 The George Institute for Global Health Australia, UNSW, PO Box M201, Missenden Rd, Sydney, NSW 2050 Australia; 2 Aboriginal and Torres Strait Islander Health, College of Medicine and Public Health, Flinders University, GPO Box 2100, Adelaide, SA 5001 Australia; 3 Watto Paruna Aboriginal Health for the Northern Adelaide Local Health Network, Corner of Mark and Oldham Roads, Elizabeth Vale, SA 5112 Australia; 4 Graduate School of Health, University of Technology Sydney, PO Box 123, Broadway, Sydney, NSW 2007 Australia; 5 Sydney Medical School, The Children’s Hospital at Westmead Clinical School, Faculty of Medicine and Health, The University of Sydney, Westmead, NSW 2145 Australia; 6 School of Public Health and Community Medicine, UNSW, Sydney, 2052 Australia

**Keywords:** Length of stay, Aboriginal and Torres Strait islander, Children, Burn injury, Prognostic factors

## Abstract

**Background:**

Aboriginal and Torres Strait Islander children have higher incidence, severity and hospital length of stay for their acute burn injuries than other Australian children. We examined factors contributing to longer length of stay for Aboriginal and Torres Strait Islander children with an acute burn injury.

**Methods:**

Burns Registry of Australia and New Zealand admissions of children < 16 years of age between October 2009 and July 2018 were analysed. Descriptive statistics explored patient and injury characteristics; Cox-regression models estimated characteristics associated with longer length of stay. Knowledge Interface methodology and Indigenous research methods were used throughout.

**Results:**

A total of 723 children were identified as Aboriginal and Torres Strait Islander and 6257 as other Australian. The median hospital length of stay for Aboriginal and Torres Strait Islander children (5 days [CI 5–6]) was 4 days longer than other Australian children (1 day [CI 1–2]). Remoteness, flame burns, high percentage total body surface area (%TBSA) and full thickness burns were factors associated with longer length of stay for Aboriginal and Torres Strait Islander children. Similar prognostic factors were identified for other Australian children along with Streptococcus sp. and Staphylococcus sp. infection.

**Conclusion:**

Remoteness, flame burns, %TBSA, and full thickness burns are prognostic factors contributing to extended hospital length of stay for all Australian children. These factors are more prevalent in Aboriginal and Torres Strait Islander children, impacting length of stay. Treatment programs, clinical guidelines, and burns policies should engage with the unique circumstances of Aboriginal and Torres Strait Islander children to mitigate inequities in health.

## Background

Aboriginal and Torres Strait Islander people, one of the longest surviving civilisations in the world, make up 3% of the Australian population: that is over 700,000 people (Australian Institute of Health and Welfare 2015; Salmon et al. 2019). Similar to other First Nation peoples globally impacted by colonisation, they face significant and ongoing health disparities, with a disease burden 2.3 times greater and life expectancy 10 years less than other Australians (Australian Institute of Health and Welfare 2015; Salmon et al. 2019). This burden is reflected in a young age profile, where 34% of Aboriginal and Torres Strait Islander people are under 15 years of age compared to 18% of other Australians (Australian Institute of Health and Welfare 2015). Further comparison to other Australian children reveals significant disadvantage across a range of social exclusion indicators, such as lower numeracy and literacy levels, financial hardship, and anxiety induced by racism (Walter 2016; Department of Social Services 2015; Priest et al. 2016; Priest et al. 2011; Commonwealth of Australia 2018). These inequities in social exclusion indicators are indicative of transgenerational trauma and ongoing marginalisation. Despite this adversity, Aboriginal and Torres Strait Islander children remain strong and resilient, demonstrating higher levels of social consciousness (compassion, empathy and caring) over other Australian children (Department of Social Services 2015; Priest et al. 2016).

Injury is a significant cause of morbidity in Aboriginal and Torres Strait Islander children (Australian Institute of Health and Welfare 2015; Department of Social Services 2009). Specifically, burn injury rates are 2–3 times greater for Aboriginal and Torres Strait Islander children as compared to other Australian children (Ivers et al. 2015; Duke et al. 2011). They also represent one of the most common injuries requiring medical attention for Aboriginal and Torres Strait Islander children < 6 years of age (Department of Social Services 2009; Department of Social Services 2011). As an injury burns are complex, resource intensive and costly, creating significant occupation and social limitations (Kornhaber et al. 2017; Dolp et al. 2018; Weedon and Potterton 2011; McGarry et al. 2014). Depending on severity, children and their families can have extensive hospital lengths of stay (LOS) for initial treatment, as well as ongoing rehospitalisation for treatment and scar management. The average hospital LOS in Aboriginal and Torres Strait Islander children hospitalised for asthma, chest or skin infections is 2–5 nights; for burn injuries it is 6 nights (Department of Social Services 2009; Department of Social Services 2011; Möller et al. 2017). Long admissions can cause significant trauma, instigating episodes of posttraumatic stress in parents, which also impacts adversely on a child’s recovery and health outcomes (McGarry et al. 2015; Moore et al. 2015). These are factors which can be exacerbated in Aboriginal and Torres Strait Islander families, who already face high levels of trauma, and who typically place less trust in the hospital system than their general practitioner (Department of Social Services 2012).

Internationally there is a sound body of knowledge surrounding prognostic factors for hospital LOS in burn injuries (Dolp et al. 2018; Moore et al. 2015; Hussain and Dunn 2013; AbdelWahab et al. 2018; Taylor et al. 2017; Gravante et al. 2007; Sierra Zúñiga et al. 2016; Matin et al. 2015; Elrod et al. 2019; Wang et al. 2018). In adults, percentage total body surface area (%TBSA), full thickness burns, socioeconomic status (SES), age, inhalation injury, comorbidities, surgical interventions, and injury complications have been shown to be associated with a longer LOS (Dolp et al. 2018; Moore et al. 2015; Hussain and Dunn 2013; AbdelWahab et al. 2018; Taylor et al. 2017; Gravante et al. 2007; Sierra Zúñiga et al. 2016). In children with a burn injury, large %TBSA (> 16%), burn cause (scald or flame compared to other types), full and partial thickness burns increase LOS, whereas younger age and early skin grafting decrease LOS (Matin et al. 2015; Elrod et al. 2019; Wang et al. 2018). There has been comparatively limited research focussed on understanding prognostic factors contributing to longer LOS for burn injury in First Nation children. Most Australian burns research has focussed on children or adults, without exploration of outcomes for Aboriginal and Torres Strait Islander people. To date, this burns injury enquiry has lacked contextualisation and appropriate analyses for Aboriginal and Torres Strait Islander communities. These processes inhibit development of appropriate care pathways and prioritisation of Indigenous approaches to health and well-being for burns injury. Therefore, our objectives were to engage with Indigenous research methodologies to identify prognostic factors of LOS in acute burn injuries for Aboriginal and Torres Strait Islander children and examine contributing health inequities.

## Methods

The Burns Registry of Australian and New Zealand (BRANZ) captures data from 17 specialist burns centres in Australia and New Zealand, with 11 of these contributing paediatric data (Riedlinger et al. 2015; Tracy et al. 2017; BRANZ 2018). With sole purpose to monitor burns incidence and causality, the BRANZ is described as a clinical epidemiological repository (Tracy et al. 2017). The registry includes patients admitted within 28 days of their injury, burn transfers, and cases managed by dedicated burn units (Riedlinger et al. 2015; Tracy et al. 2017; BRANZ 2018). The BRANZ was established before the full conceptualisation and appreciation of Indigenous data sovereignty, and as such does not have Aboriginal and Torres Strait Islander governance surrounding status identification, data collection, data analyses or outcome translation (Walter 2018; Kukutai and Taylor 2016). We therefore employed decolonising and knowledge interface methodologies in this study (Supplementary File 1 – Fig. A) (Kukutai and Taylor 2016; Walter and Andersen 2013; Martin 2003; Sherwood 2013; Sherwood and Edwards 2006). Indigenous knowledges were important for the overall study conceptualisation (variable selection, statistical analyses, results interpretation) and the first author drew on their own lived experience as an Aboriginal woman, and engaged in yarning and yuri ingarninthii^1^ processes with other experts (Aboriginal Public Health Physician A/Prof Tamara Mackean), Registered Aboriginal Health Practitioner specialised in burns (Kurt Towers) to strengthen this process (Supplementary File 1 - Fig. A) (Bessarab and Ng'andu 2010; Walker et al. 2014).

### Data source

The BRANZ data included in this study were any records with admission between 01 October 2009 and 31 July 2018, for Australian children (< 16 years) with an acute burns admission to an Australian hospital. Data was extracted under the following categories (Variable Details – Supplementary File 2 – Table B):

1. Demographics: Gender, Age, Accessibility/Remoteness Index of Australia (ARIA), and SES.
2. Injury Severity: burn cause, %TBSA, Burn Depth, and Bacterial infection.
3. Injury treatment: Burn Dressing, Burn Debridement, Split Skin Graft, Physio/Occupational Therapy, Psychology/Social Work, and Other Allied Health (Supplementary File 2 - Table B).

### Statistical analysis

Descriptive statistics were used to describe patient demographics and injury and treatment characteristics. Continuous variables were summarised with mean, standard deviation, and median. Categorical variables were summarised as the proportion of the patients for each level of the variable. Missing data on patient characteristics were imputed in a multiple imputation procedure using chained equations with predictive mean matching over 40 imputations (Morris et al. 2014). Crude hospital LOS was represented visually through inverse survival hazard estimates in both child groups (with 95% CI), and the median time to discharge (with 95% CI) was estimated.

A four staged Cox regression model, stratified by Aboriginal and Torres Strait Islander status, was developed to examine factors which increased hospital LOS for both Aboriginal and Torres Strait Islander children and for other Australian children. Hazard ratio outputs from the model, where survival time was hospital LOS, were described as the discharge ratio^2^ (95% CI), in the model. Higher discharge ratios reflect longer LOS. Each stage introduced new variables to the model:

1. Stage 1 (Table 1): Patient Demographics: Gender, Age Range, ARIA and SES
2. Stage 2 (Table 2): Injury Cause
3. Stage 3 (Table 3): Injury Severity: %TBSA, Depth, & Wound Bacterial Infection
4. Stage 4 (Table 4): Injury Treatment: Burn Dressing, Burn Debridement, Split Skin Graft, and Allied Health Intervention.

**Table 1:** Discharge (hazard) ratio to Patient Demographics (Gender, Age Range, ARIA and SES)

Aboriginal & Torres Strait Islander Children				
Discharge Ratio:	Stage 1 Patient characteristics	Stage 2 Injury Cause	Stage 3 Injury Severity	Stage 4 Treatment
**Gender**				
Male	1.1 (0.9–1.3)	1.1 (0.9–1.2)	**1.1 (1.0–1.3)**	**1.2 (1.0–1.4)**
Female	Reference	Reference	Reference	Reference
**Age Range**				
Less than 1 year	1.1 (0.8–1.6)	1.4 (1.0–2.0)	1.0 (0.7–1.4)	1.00 (0.7–1.4)
1 to 4 years	0.9 (0.7–1.1)	1.0 (0.8–1.2)	0.9 (0.7–1.1)	0.9 (0.7–1.1)
5 to 9 years	1.1 (0.9–1.4)	1.1 (0.8–1.4)	0.9 (0.7–1.2)	0.9 (0.7–1.2)
10 to 15 years	Reference	Reference	Reference	Reference
**Remote Area Index**				
Metropolitan (RA1)	Reference	Reference	Reference	Reference
Inner Regional (RA2)	1.0 (0.8–1.3)	1.1 (0.8–1.4)	1.0 (0.7–1.3)	1.0 (0.7–1.3)
Outer Regional (RA3)	**1.3 (1.0–1.6)**	**1.3 (1.1–1.6)**	**1.5 (1.2–1.9)**	**1.8 (1.4–2.3)**
Remote (RA4)	**1.7 (1.3–2.2)**	**1.8 (1.4–2.3)**	**1.9 (1.5–2.5)**	**2.4 (1.9–3.2)**
Very Remote (RA5)	**1.6 (1.2–2.1)**	**1.7 (1.3–2.3)**	**2.0 (1.5–2.7)**	**2.6 (1.9–3.5)**
**Index of Relative Socio-Economic Advantage and Disadvantage**				
Very Low (1, 2)	1.2 (0.9–1.7)	1.2 (0.8–1.6)	0.9 (0.7–1.3)	0.9 (0.6–1.3)
Low (3, 4)	1.1 (0.8–1.6)	1.1 (0.8–1.6)	0.9 (0.6–1.3)	0.9 (0.6–1.3)
Middle (5, 6)	1.0 (0.7–1.4)	0.9 (0.6–1.3)	0.8 (0.5–1.1)	0.8 (0.5–1.1)
High (7, 8)	1.1 (0.7–1.6)	1.0 (0.7–1.5)	0.8 (0.5–1.1)	0.8 (0.5–1.2)
Very High (9, 10)	Reference	Reference	Reference	Reference
**Other Australian Children**				
**Gender**				
Male	1.0 (0.9–1.0)	1.0 (0.9–1.0)	0.9 (0.9–1.00)	1.0 (0.9–1.0)
Female	Reference	Reference	Reference	Reference
**Age Range**				
Less than 1 year	0.9 (0.8–1.0)	1.0 (0.9–1.2)	1.0 (0.9–1.1)	1.0 (0.9–1.1)
1 to 4 years	0.9 (0.8–1.0)	1.0 (0.9–1.1)	1.0 (0.9–1.0)	0.9 (0.9–1.0)
5 to 9 years	1.0 (0.9–1.1)	1.0 (1.0–1.1)	1.0 (0.9–1.1)	1.0 (0.9–1.1)
10 to 15 years	Reference	Reference	Reference	Reference
**Remote Area Index**				
Metropolitan (RA1)	Reference	Reference	Reference	Reference
Inner Regional (RA2)	**1.1 (1.1–1.2)**	**1.2 (1.1–1.3)**	**1.2 (1.1–1.3)**	**1.2 (1.1–1.3)**
Outer Regional (RA3)	**1.4 (1.3–1.5)**	**1.5 (1.3–1.6)**	**1.6 (1.4–1.7)**	**1.5 (1.4–1.7)**
Remote (RA4)	**1.6 (1.3–1.9)**	**1.7 (1.5–2.1)**	**1.9 (1.6–2.2)**	**1.9 (1.6–2.3)**
Very Remote (RA5)	**1.6 (1.2–2.1)**	**1.9 (1.5–2.5)**	**2.2 (1.7–2.9)**	**2.2 (1.7–2.9)**
**Index of Relative Socio-Economic Advantage and Disadvantage**				
Very Low (1, 2)	**1.1 (1.0–1.2)**	1.0 (0.9–1.1)	1.0 (0.9–1.1)	0.9 (0.9–1.0)
Low (3, 4)	**1.0 (1.0–1.1)**	1.00 (0.9–1.1)	1.0 (0.9–1.1)	0.9 (0.9–1.0)
Middle (5, 6)	**1.1 (1.0–1.2)**	**1.1 (1.0–1.1)**	**1.0 (1.0–1.1)**	1.0 (0.9–1.1)
High (7, 8)	**1.1 (1.0–1.2)**	**1.1 (1.0–1.2)**	**1.1 (1.0–1.2)**	**1.1 (1.0–1.2)**
Very High (9, 10)	Reference	Reference	Reference	Reference

Discharge ratios is the hazard ratio. A discharge ratio > 1.0, signifies statistically significant longer LOS over the reference variable. Statistically significant results are bolded

**Table 2:** Discharge (hazard) ratio to Burns Injury Cause

Aboriginal & Torres Strait Islander Children			
Discharge Ratio:	Stage 2 Injury Cause	Stage 3 Injury Severity	Stage 4 Treatment
**Primary Burn Cause**			
Scald	Reference	Reference	Reference
Contact	0.9 (0.7–1.0)	1.0 (0.8–1.2)	1.0 (0.8–1.2)
Flame	**1.6 (1.3–2.0)**	**1.3 (1.0–1.6)**	**1.3 (1.0–1.6)**
Other	1.0 (0.7–1.3)	1.0 (0.7–1.4)	1.1 (0.8–1.5)
**Other Australian Children**			
**Primary Burn Cause**			
Scald	Reference	Reference	Reference
Contact	0.7 (0.7–0.8)	0.8 (0.8–0.9)	0.9 (0.8–1.0)
Flame	**1.6 (1.5–1.8)**	**1.1 (1.0–1.2)**	**1.1 (1.0–1.2)**
Other	0.8 (0.7–0.9)	0.9 (0.8–0.9)	1.0 (0.9–1.0)

Other: electrical, chemical, no cause recorded

**Table 3:** Discharge (hazard) ratio to Injury Severity (%TBSA, Depth, & Wound Bacterial Infection)

Aboriginal & Torres Strait Islander Children			Other Australian Children	
Discharge Ratio:	Stage 3 Injury Severity	Stage 4 Treatment	Stage 3 Injury Severity	Stage 4 Treatment
**% Total Body Surface Area Range**				
Below 10%	Reference	Reference	Reference	Reference
10 to 19%	**2.6 (1.9–3.5)**	**2.2 (1.6–3.1)**	**2.2 (2.0–2.5)**	**2.0 (1.8–2.2)**
Greater than 19%	**5.1 (3.1–8.3)**	**4.0 (2.3–7.0)**	**5.3 (4.4–6.4)**	**5.0 (4.0–6.1)**
**Burn Depth**				
Superficial Thickness	Reference	Reference	Reference	Reference
Partial Thickness	1.3 (0.9–1.7)	1.1 (0.8–1.5)	0.8 (0.7–0.9)	0.8 (0.7–0.9)
Full Thickness	**2.0 (1.4–2.9)**	**1.8 (1.2–2.6)**	1.0 (0.9–1.2)	**1.1 (1.0–1.3)**
**Bacterial Infection**				
No	Reference	Reference	Reference	Reference
Streptococcus	1.3 (0.9–1.8)	1.2 (0.8–1.7)	**1.5 (1.3–1.8)**	**1.4 (1.2–1.7)**
Staphylococcus	**1.4 (1.1–1.8)**	1.2 (0.9–1.5)	**1.4 (1.3–1.5)**	**1.3 (1.1–1.4)**

**Table 4:** Discharge (hazard) ratio to Treatment (Burn Dressing, Burn Debridement, Split Skin Graft, and Allied Health Intervention)

Discharge Ratio:	Stage 4 Treatment	
	Aboriginal and Torres Strait Islander	Other Australian
**Burn Dressing**		
No	Reference	Reference
Less than 10%	**1.4 (1.0–1.9)**	**1.1 (1.0–1.2)**
Greater than 10%	1.4 (0.9–2.3)	1.0 (0.9–1.2)
**Sedation**		
No	Reference	Reference
Yes	0.9 (0.6–1.2)	1.0 (0.9–1.1)
**Burn Debridement**		
No	Reference	Reference
Non-excisional	**1.5 (1.0–2.2)**	**1.3 (1.2–1.5)**
Excisional	**1.3 (1.0–1.8)**	**1.3 (1.2–1.4)**
**Split Skin Graft**		
No	Reference	Reference
Less than 3%	**1.6 (1.1–2.2)**	1.0. (0.9–1.1)
Greater than 3%	**2.0 (1.5–2.8)**	**1.6 (1.5–1.8)**
**Allied Health Intervention**		
No	Reference	Reference
Yes	**1.6 (1.3–2.1)**	**2.2 (2.1–2.4)**

Allied Health: physiotherapy, occupational therapy, social work, psychology, dietetics, speech pathology, audiology, prosthetics, orthotics, pharmacy

The proportional hazards assumption for each variable was checked on the first imputation of the dataset using visualisations of the cumulative sum of martingale residuals over follow-up. Data were prepared, analysed, and plotted with Stata 15.1 (StataCorp, Texas, TX USA) and SAS/Stat 14.2 (SAS Institute, Cary, NC, USA).

### Ethics approval

The Human Research Ethics Committee at the University of New South Wales of Sydney (HC17712) and Aboriginal Health & Medical Research Council of New South Wales (1032/14) approved ethics for this study.

## Results

### Patient demographics and injury severity

From 01 October 2009 to 31 July 2018, 6980 children were admitted to a BRANZ reporting hospital for an acute burns injury. Of this cohort 10.4% (CI 9.7–11.1) were recorded as an Aboriginal and/or Torres Strait Islander child (Supplementary File 3 – Table C). The median LOS in hospital for Aboriginal and Torres Strait Islander children (5 days [CI 5–6]) was 4 days longer than other Australian children (1 day [CI 1–1]). Aboriginal and Torres Strait Islander children in the registry were more likely to be male (61.8%), children ≤4 years of age (60.0%), live in outer regional areas (28.9%), from very low SES backgrounds (51.2%), and with burns caused from scalds(39.4%). For these children partial thickness burns (75.7%) and %TBSA below 10 (84.7%) were more prevalent, with common treatments including split skin grafts above 3% body surface area (%BSA)(10.9%), excisional debridement (12.2%) and social work or psychology intervention (36.4%)(Supplementary File 3 – Table C). Other Australian children in the registry had similar demographics and injury severity, however higher proportions of burns were apparent in other Australian children residing in metropolitan locations (61.0%), from very low SES backgrounds (25.1%), primary burns cause of scalds (51.0%), common treatment of split skin grafts below 3%BSA (11.9%), and social work or psychology intervention (25.8%).

### Prognostic factors

#### Survival Hazard estimates

**Fig. 1:**
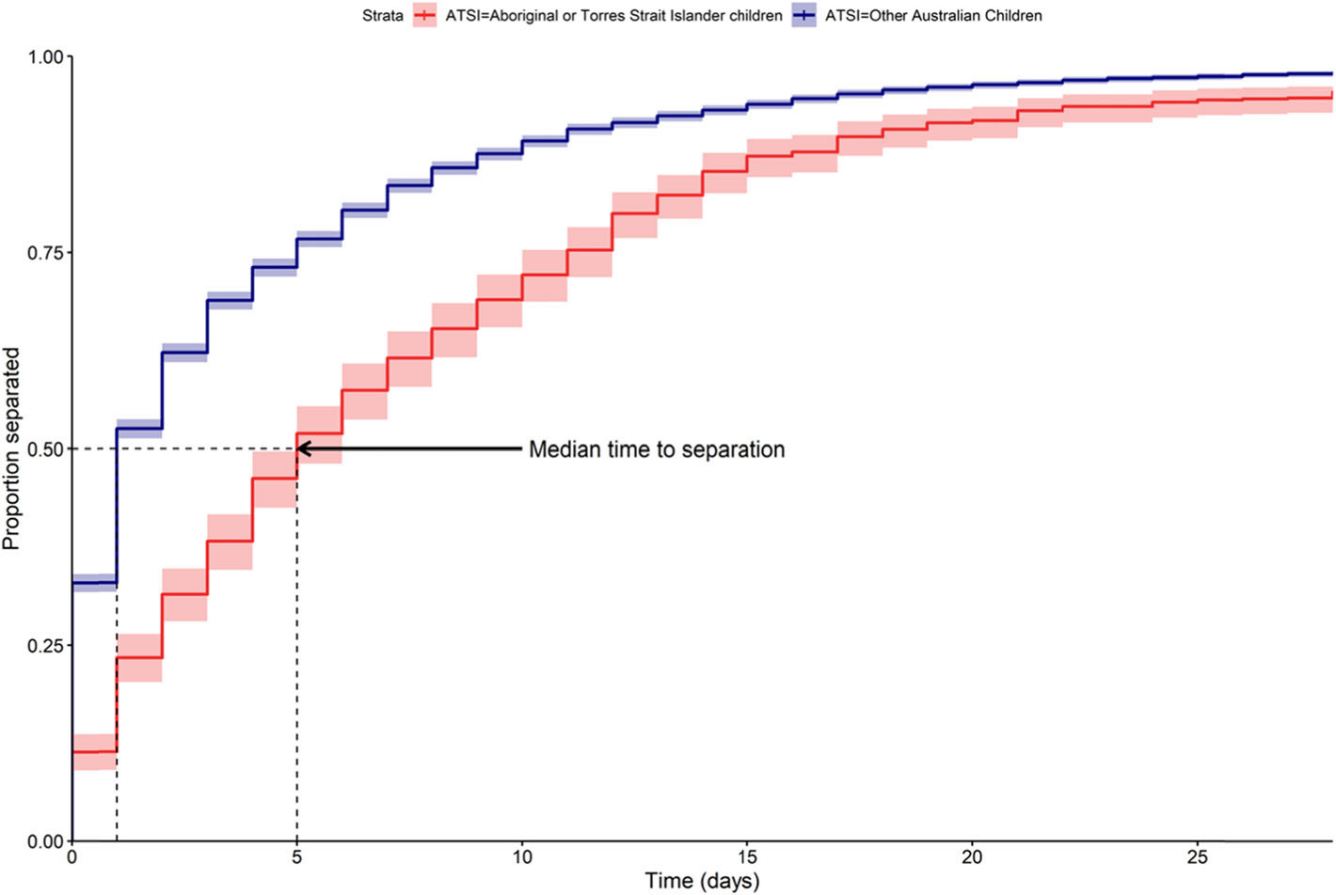
Kaplan-Meier estimates for hospital discharge across both child groups

Inverse survival hazards estimates (Fig. 1) demonstrated Aboriginal and Torres Strait Islander children remain in hospital for longer periods as compared to other Australian children.

#### Staged cox-regression model

Age and socioeconomic status (Stage 1) had no impact on LOS for Aboriginal and Torres Strait Islander children, across all stages of the model (Table 1). Male gender, injury severity, and injury treatment were associated with greater hospital LOS for Aboriginal and Torres Strait Islander patients. Aboriginal and Torres Strait Islander children from outer regional to very remote areas had 30–60% longer hospital LOS than metropolitan children, with discharge ratios of 1.3 (CI 1.0–1.6) to 1.6 (CI 1.2–2.1). When adding injury cause, severity and treatment to the model, LOS increased for these children, with discharge ratios of 1.8 (CI 1.4–2.3) to 2.6 (CI 1.9–3.5). Similarly, remote ARIA residency increased LOS for other Australian children, although metropolitan residency was associated with reduced LOS, as compared to inner regional to very remote residency, across all model stages (Table 1). Other Australian children who resided in high SES areas had slightly longer LOS across all model stages as compared to children in very high SES areas, for residence in middle SES regions an elevated LOS was evident for injury cause and severity.

Flame, as compared to scald burns (Stage 2), had the highest discharge ratio for both Aboriginal and Torres Strait Islander children and other Australian children (Table 2). This significance remained even after controlling for injury severity and treatment.

For factors related to injury severity (Stage 3), presence of Staphylococcus sp. infection, full thickness burns and %TBSA increased LOS for Aboriginal and Torres Strait Islander children (Table 3). After controlling for treatment, full thickness burns and %TBSA remained as significant factors in the model. The greatest discharge ratio for Aboriginal and Torres Strait Islander children was %TBSA, with a discharge ratio of 2.6 (CI 1.9–3.5) for burn 10–19%TBSA, and 5.1 (CI 3.1–8.3) for burns greater than 19%TBSA, as compared to burns less than 10%TBSA. Discharge ratios did decrease for %TBSA after controlling for different treatments. Similar discharge ratios were evident in other Australian children for %TBSA. Both Streptococcus sp. and Staphylococcus sp. infections significantly increased discharge ratios for other Australian children, which remained after controlling for treatment (Table 3).

Factors relating to treatment (Stage 4), including allied health intervention, burn dressings less than 10%TBSA, any burn debridement or split skin graft were significantly associated with discharge ratios for Aboriginal and Torres Strait Islander children (Table 4). The highest treatment discharge ratio for Aboriginal and Torres Strait Islander children was for split skin grafts above 3%BSA (2.0 [CI 1.5–2.8]), as compared to children without this treatment. Similar results were evident in other Australian children, with the greatest discharge ratio found in children who had an allied health intervention (2.2 [2.1–2.4])(Table 4).

## Discussion

In this study, Aboriginal and Torres Strait Islander children had a median hospital LOS of 5 days, which is longer than the 4 days previously reported (Duke et al. 2011; Tracy et al. 2017; Dyson et al. 2015; de Silva et al. 2014). We also found that Aboriginal and Torres Strait Islander children had a hospital LOS 4 days longer than other Australian children, compared to past reporting of 3 days (Duke et al. 2011; Möller et al. 2017; Tracy et al. 2017; Dyson et al. 2015; de Silva et al. 2014). These extra days of hospital stay represent additional burden on Aboriginal and Torres Strait Islander families not previously captured, be that through out-of-pocket healthcare expenditure, treatment severity, isolation, re-traumatisation, strain on family connections, and may be associated with poorer function and health-related quality of life (Ryder et al. 2019).

Aboriginal and Torres Strait Islander children from very remote locations had longer hospital LOS than metropolitan children, and LOS was also impacted by primary burn cause (flame versus scald), greater injury severity, and various treatment factors (split skin graft, burn debridement, allied health intervention). Similar findings were observed in other Australian children, however the proportion of children residing in rural and remote locations and discharge ratios were greater overall for Aboriginal and Torres Strait Islander children. Longer hospital LOS for burns injury admissions has previously been reported in rural and remote Australia, and a range of factors are likely to contribute to this, such as access to appropriate health services (Möller et al. 2017; Hyland et al. 2015; Humphreys 2009). In these settings, for Aboriginal and Torres Strait Islander people and other Australians, major challenges remain in the delivery of healthcare including high costs, limited resources, varying staff retention, training access and expertise level with most rural and remote clinical staff trained as generalists (Humphreys 2009; Wakerman et al. 2008; Russell et al. 2013). These factors may all directly impact on both first aid and treatment for serious burn injuries.

The majority of remote communities are reliant on small local health centres, general practices and/or community controlled or Aboriginal specific services. Such local services may consist of on-call nursing staff and Aboriginal Health Workers, who may be the main point of call for several communities over vast geographical locations. These services are restricted in the treatment and expertise they can provide for serious burns. Serious burns require air retrieval or ambulance transfer to more specialised tertiary facilities. These transport services are impacted by availability, weather conditions and other factors. Additionally, decreased capacity to access appropriate burns first aid treatment has been reported in remote Australia (Read et al. 2018). In these settings access to clean cool running water can be limited by inadequate community infrastructure. All of these elements disadvantage access to best practice treatment options for serious burns. For instance, skin substitute Biobrane™ has an optimal application window from time of burn occurrence (Harish et al. 2019; Greenwood et al. 2009).

Issues of remoteness may be further compounded by community and cultural obligations. For example, Aboriginal and Torres Strait Islander families may travel away from home or to remote locations for sorry business,^3^ visiting communities or camping where running water may not exist (Read et al. 2018). We found that flame burns increased hospital LOS for Aboriginal and Torres Strait Islander children. Compared to other causative mechanisms, flame burns typically are of greater thickness, risk of infection and severity, requiring intense and invasive surgical intervention which increases LOS. Past reporting has found a higher incidence of more invasive treatment i.e. skin grafting, in Australian children from rural locations as compared to metropolitan areas (Hyland et al. 2015). We found that split skin grafts increased LOS for Aboriginal and Torres Strait Islander children in this study, which is different to past research in other populations, which reported grafting as decreasing LOS in children (Wang et al. 2018). Recent research has suggested that Aboriginal and Torres Strait Islander children are remaining longer in hospital for their burns treatment, due to perceived social issues from clinical teams and for easier access to multidisciplinary support and care i.e. allied health support (Fraser et al. 2019). However, while we found allied health intervention was proportionally greater in Aboriginal and Torres Strait Islander children, higher discharge ratios were found in other Australian children with allied health intervention, which is at odds with the rationale that LOS is increased to improve access to multidisciplinary support. These outcomes are also different to recent poisoning research, suggesting similar allied health contact levels between Aboriginal and other Australian children (Lee et al. 2019).

The most significant prognostic factor providing the highest discharge ratios in all children was %TBSA, similar to previous research (Dolp et al. 2018; Möller et al. 2017; Hussain and Dunn 2013; AbdelWahab et al. 2018; Taylor et al. 2017; Gravante et al. 2007; Matin et al. 2015; Elrod et al. 2019; Wang et al. 2018; Hyland et al. 2015; Meshulam-Derazon et al. 2006). In Aboriginal and Torres Strait Islander children, greater %TBSA levels have been reported to increase LOS, however our ratios were greater than previous reports (Möller et al. 2017). Full thickness burns was another prognostic factor for Aboriginal and Torres Strait Islander children, again in line with past reporting (Möller et al. 2017). In adult populations, full thickness burns and bacterial infections have been associated with increased LOS (Dolp et al. 2018; Hussain and Dunn 2013; AbdelWahab et al. 2018; Taylor et al. 2017; Gravante et al. 2007; Wang et al. 2018). Although Aboriginal and Torres Strait Islander children had a higher proportion of bacterial infection, it was not associated with increased LOS, unlike for other Australian children. It is possible that more Aboriginal children have bacterial infection as an underlying comorbidity, potentially representing differentials in environment and living conditions (ie. poverty), or barriers accessing health services or provision of culturally responsive healthcare treatment (Indigenous Allied Health Association 2015). This can have significant impacts for health later in life, including renal disease (Cass et al. 2004).

There are also additional factors which could contribute to increases in hospital LOS for Aboriginal and Torres Strait Islander children, which BRANZ does not currently record. There is a ‘remote-alisation’ and over medicalisation of Aboriginal and Torres Strait Islander children (Fraser et al. 2019; Waddell and Dibley 1986; Ryder et al. 2017) where the Australian healthcare system inadvertently fosters implicit bias, systemic racism, power imbalances and racialisation of Aboriginal and/or Torres Strait Islander identity with presumptive beliefs on remoteness (Fraser et al. 2019; Waddell and Dibley 1986; Ryder et al. 2017; Shahid et al. 2013; Durey and Thompson 2012; Sherwood 2009). These presumptive beliefs modify the diagnostic reasoning process for clinicians when working with Aboriginal and Torres Strait Islander children and their families (Fraser et al. 2019; Waddell and Dibley 1986; Ryder et al. 2017) and may potentially (and unintentionally) result in longer hospital LOS in Aboriginal and Torres Strait Islander children with a burns injury (Fraser et al. 2019; Waddell and Dibley 1986). Further, burns care continues to be based on Western biomedical paradigms, predominantly focussing on the clinical characteristics of the burn injury with less emphasis on the psychosocial aspects (Fraser et al. 2019; Alonso 2004; Baum 2016). This is disconnected to Aboriginal and Torres Strait Islander models of health and well-being, which are holistic and interconnected, encompassing the child, their family and community (Salmon et al. 2019; Walter 2016; Priest et al. 2016; Priest et al. 2012; Milroy 2008). Clinical teams should work with families in regards to their needs and obligations, with support from Aboriginal Healthcare Workers and local health services, so that hospital LOS for treatment can be appropriately managed. Collaborative, family-centred actions such as this, act to alleviate stress and burden on Aboriginal and Torres Strait Islander families, which in turn facilitates a child achieving wellbeing (Priest et al. 2012; Purdie et al. 2012; Zubrick et al. 2004).

In this study rural residency, burn severity (flame, high %TBSA, full thickness burns) and complex treatment (split skin grafts) are prognostic factors contributing to increased hospital LOS for Aboriginal and Torres Strait Islander children. These factors are also health inequities, as greater proportions of Aboriginal and Torres Strait Islander children are impacted, compared to other Australian children. Remote Aboriginal and Torres Strait Islander children who suffer a complex burn have reduced or delayed access to healthcare for burns treatment and rehabilitation, creating longer hospital LOS. Adding in factors such as low SES, also more prevalent in this cohort, is likely to lead to additional financial burden from out-of-pocket healthcare expenses, which can create isolation and impact on recovery (Callander et al. 2019; Hynd et al. 2008; Jan et al. 2011; Couzos and Murray 2008; Couzos 2005). Such factors can be further compounded by culturally unsafe healthcare, which creates a lack of autonomy or self-determination in the treatment and decision making process for Aboriginal and Torres Strait Islander families (Fraser et al. 2019). Specialised burn services should actively work to counteract LOS as a health inequity manifestation, through staff training in cultural safety and reflexivity as a minimum standard, along with tertiary services establishing quality indicators of cultural safety (Durey et al. 2012). Review of government policies and initiatives designed to address the needs of rural Aboriginal and Torres Strait Islander patients, such as the Patient Assisted Travel Scheme may also be warranted, with consideration of unavoidable excessive LOS for patients of low SES.

### Strengths & Limitations

Strengths include our use of 9 years of national data, with a focus on LOS prognostic factors for Aboriginal and Torres Strait Islander children. Our use of Indigenous research methodology and methods (Supplementary File 1 - Fig. A) approach to missing data and focus on health inequities provides a greater appreciation of the depth and breadth of burn injuries present in Aboriginal and Torres Strait Islander children.

This analysis has some limitations. SES was defined based on neighbourhood postcode and may not be a true representation (Moore et al. 2015). BRANZ captures data from reporting hospitals only (generalist hospitals are not included) and not all hospitals have been reporting for the same length of time. BRANZ is a western biomedically constructed, epidemiological repository where colonial constraints and whiteness are present and unacknowledged (Kukutai and Taylor 2016; Walter and Andersen 2013). Under-identification of Aboriginal and Torres Strait Islander children is not unusual in registries of this type (Möller et al. 2017; Randall et al. 2013). Further to this, the BRANZ uses International Classification of Disease codes, which do not code for Aboriginal Liaison Officers, Aboriginal Health Workers, Aboriginal Health Practitioners, or Aboriginal Traditional Healers, despite coding for other interventions such as ‘Spiritual Care’ under ‘Allied Health’ (Australian Consortium for Classification Development 2017). If these data were recorded it would provide significant insight into the relationship between Aboriginal and Torres Strait Islander healthcare practice and models of care with hospital LOS. In order to capture data that appropriately records factors impacting on patient outcomes, data registries should engage with Aboriginal and Torres Strait Islander health bodies to broaden coding, to capture community needs and work towards data sovereignty. Data which recognises and responds to the health and well-being concepts and needs of Australia’s First Peoples is a step towards data sovereignty for Aboriginal and Torres Strait Islander communities, (Walter 2018; Walter and Suina 2019). An exemplar of this include the Footprints in Time Study, with Indigenous leadership, oversight, and a focus on positive strength based data collection and reporting (Salmon et al. 2019).

## Conclusion

Remoteness, %TBSA, flame and full thickness burns are prognostic factors creating greater LOS in hospital burns admissions for Aboriginal and Torres Strait Islander children. This health inequity introduces additional and unique burdens on these families including financial hardship, isolation and lack of autonomy. Burdens which can be exacerbated by racism and whiteness, thus impacting negatively on an Aboriginal and/or Torres Strait Islander child’s wellbeing. The complexities and uniqueness of these burdens need to be considered in burns data repositories, clinical models of care, policies and targeted burns interventions for prevention and treatment in Aboriginal and Torres Strait Islander communities. Aboriginal and Torres Strait Islander families experiencing significant hospital LOS warrant further support (emotionally, financially or socially), to decrease the risk of further marginalisation.

## Supplementary information

**Additional file 1 Fig. A.** Knowledge interface methodology, comprising of Indigenous and Western knowledge system for this study.

**Additional file 2 Table B.** Variables extracted and created from data source with their definitions.

**Additional file 3.** Table C: Patient characteristics.

## Data Availability

The data that support the findings of this study are available from Burns Registry of Australian and New Zealand but restrictions apply to the availability of these data, which were used under license for the current study, and so are not publicly available. Data are however available from the authors upon reasonable request and with permission of Burns Registry of Australian and New Zealand.

## Abbreviations

LOS: Length of Stay
SES: Socio Economic Status
BRANZ: Burns Registry of Australia and New Zealand
ARIA: Accessibility/Remoteness Index of Australia
PROGRESS-Plus: Place Residence, Race/Ethnicity, Occupation, Gender, Religion, Education, Social-Capital, Socioeconomic Position, Age, Disability, Sexual Orientation, Other Vulnerable Groups
%TBSA: Percentage Total Body Surface Area
%BSA: Percentage Body Surface Area
CI: Confidence Interval

## Footnotes

1 Yarning is an Indigenous research method, a conversational technique for data collection and discussion. Yuri ingarninthii, a Kaurna term, is a form of deep listening. Both are used in Indigenous research.

2 Discharge Ratio – Hazard Ratio for survival time, where survival time is LOS.

3 Sorry business – cultural, spiritual and social responsibilities, obligations and ceremonies associated with death in Aboriginal and Torres Strait Islander communities.

## References

[CR1] AbdelWahab ME, Sadaka MS, Elbana EA, Hendy AA (2018). Evaluation of prognostic factors affecting length of stay in hospital and mortality rates in acute burn patients. Ann Burns Fire Disasters.

[CR2] Alonso Y (2004). The biopsychosocial model in medical research: the evolution of the health concept over the last two decades. Patient Educ Couns.

[CR3] Australian Consortium for Classification Development (2017). The international statistical classification of diseases and related health problems, tenth revision, Australian modification (ICD-10-AM/ACHI/ACS).

[CR4] Australian Institute of Health and Welfare (2015). The health and welfare of Australia’s Aboriginal and Torres Strait Islander peoples 2015.

[CR5] Baum F (2016). The new public health.

[CR6] Bessarab D, Ng'andu B (2010). Yarning about yarning as a legitimate method in indigenous research. Int J Crit Indigenous Stud.

[CR7] BRANZ (2018). Burns registry of Australia and New Zealand data dictionary.

[CR8] Callander EJ, Fox H, Lindsay D (2019). Out-of-pocket healthcare expenditure in Australia: trends, inequalities and the impact on household living standards in a high-income country with a universal health care system. Heal Econ Rev.

[CR9] Cass A, Cunningham J, Snelling P, Wang Z, Hoy W (2004). Exploring the pathways leading from disadvantage to end-stage renal disease for indigenous Australians. J Soc Sci Med.

[CR10] Commonwealth of Australia (2018). Department of the Prime Minister and Cabinet, closing the gap: prime minister report 2018.

[CR11] Couzos S (2005). PBS medications: improving access for Aboriginal and Torres Strait islander peoples. Aust Fam Physician.

[CR12] Couzos S, Murray RM (2008). Aboriginal primary health care.

[CR13] de Silva H, Gabbe B, Callaghan J, Liman J. Burns Registry of Australia and New Zealand Annual Report July 2013 – June 2014. Branz: Branz, Monash University; 2014. Report No.: 5th Annual Report.

[CR14] Department of Social Services (2009). Longitudinal study of indigenous children – key summary report from wave 1.

[CR15] Department of Social Services (2011). Longitudinal study of indigenous children – key summary report from wave 2.

[CR16] Department of Social Services (2012). Longitudinal study of indigenous children – key summary report from wave 3.

[CR17] Department of Social Services (2015). Longitudinal study of indigenous children – key summary report from wave 5.

[CR18] Dolp R, Rehou S, McCann MR, Jeschke MG (2018). Contributors to the length-of-stay trajectory in burn-injured patients. Burns..

[CR19] Duke J, Wood F, Semmens J, Edgar DW, Spilsbury K, Hendrie D (2011). A study of burn hospitalizations for children younger than 5 years of age: 1983–2008. Pediatrics..

[CR20] Durey A, Thompson SC (2012). Reducing the health disparities of indigenous Australians: time to change focus. BMC Health Serv Res.

[CR21] Durey A, Wynaden D, Thompson SC, Davidson PM, Bessarab D, Katzenellenbogen JM (2012). Owning solutions: a collaborative model to improve quality in hospital care for Aboriginal Australians. Nurs Inq.

[CR22] Dyson K, Cameron W, Gabbe B, Thomas T (2015). Burns Registry of Australia and New Zealand Annual Report July 2015–June 2016.

[CR23] Elrod J, Schiestl CM, Mohr C, Landolt MA (2019). Incidence, severity and pattern of burns in children and adolescents: an epidemiological study among immigrant and Swiss patients in Switzerland. Burns..

[CR24] Fraser S, Grant J, Mackean T, Hunter K, Keeler N, Clapham K (2019). What informs care? Descriptions by multidisciplinary teams about burns care for Aboriginal and Torres Strait islander children. Burns..

[CR25] Gravante G, Delogu D, Esposito G, Montone A (2007). Analysis of prognostic indexes and other parameters to predict the length of hospitalization in thermally burned patients. Burns..

[CR26] Greenwood JE, Clausen J, Kavanagh S (2009). Experience with biobrane: uses and caveats for success. Eplasty.

[CR27] Harish V, Li Z, Maitz PKM (2019). The optimal timing of outpatient Biobrane™ application for superficial and mid dermal partial thickness burns: evidence for the ‘12-hour rule’. Burns..

[CR28] Humphreys JS (2009). Key considerations in delivering appropriate and accessible health care for rural and remote populations: discussant overview. Aust J Rural Health.

[CR29] Hussain A, Dunn KW (2013). Predicting length of stay in thermal burns: a systematic review of prognostic factors. Burns..

[CR30] Hyland EJ, Zeni G, Harvey JG, Holland A (2015). Rural and metropolitan pediatric burns in New South Wales and the Australian Capital Territory: does distance make a difference?. J Burn Care Res.

[CR31] Hynd A, Roughead EE, Preen DB, Glover J, Bulsara M, Semmens J (2008). The impact of co-payment increases on dispensings of government-subsidised medicines in Australia. Pharmacoepidemiol Drug Saf.

[CR32] Indigenous Allied Health Association. Cultural responsiveness in action: an IAHA framework: Canberra: Indigenous Allied Health Australia; 2015.

[CR33] Ivers RQ, Hunter K, Clapham K, Coombes J, Fraser S, Lo S (2015). Understanding burn injuries in Aboriginal and Torres Strait islander children: protocol for a prospective cohort study. BMJ Open.

[CR34] Jan S, Essue BM, Leeder SR (2011). Falling through the cracks: the hidden economic burden of chronic illness and disability on Australian households. Med J Aust.

[CR35] Kornhaber R, Rickard G, McLean L, Wiechula R, Lopez V, Cleary M (2017). Burn care and rehabilitation in Australia: health professionals’ perspectives. Disabil Rehabil.

[CR36] Kukutai T, Taylor J. Indigenous data sovereignty: toward an agenda: Canberra: ANU Press; 2016. p. 79–98.

[CR37] Lee C, Hanly M, Larter N, Zwi K, Woolfenden S, Jorm L (2019). Demographic and clinical characteristics of hospitalised unintentional poisoning in Aboriginal and non-Aboriginal preschool children in New South Wales, Australia: a population data linkage study. BMJ Open.

[CR38] Martin KMB (2003). Ways of knowing, being and doing: a theoretical framework and methods for indigenous and indigenist re-search. J Aust Stud.

[CR39] Matin BK, Rezaei S, Karyani AK (2015). Factors associated with length of stay and hospital charges among pediatric burn injury in Kermanshah, west of Iran. Int J Pediatr.

[CR40] McGarry S, Elliott C, McDonald A, Valentine J, Wood F, Girdler S (2014). Paediatric burns: from the voice of the child. Burns..

[CR41] McGarry S, Elliott C, McDonald A, Valentine J, Wood F, Girdler S (2015). "this is not just a little accident": a qualitative understanding of paediatric burns from the perspective of parents. Disabil Rehabil.

[CR42] Meshulam-Derazon S, Nachumovsky S, Ad-El D, Sulkes J, Hauben DJ (2006). Prediction of morbidity and mortality on admission to a burn unit. Plast Reconstr Surg.

[CR43] Milroy H, Williams A, Cowling V (2008). Chapter 12: children are our future - understanding the needs of Aboriginal children and their families. Infants of parents with mental illness : developmental, clinical, cultural and personal perspectives.

[CR44] Möller H, Harvey L, Falster K, Ivers R, Clapham KF, Jorm L (2017). Indigenous and non-indigenous Australian children hospitalised for burn injuries: a population data linkage study. Med J Aust.

[CR45] Moore L, Cisse B, Kuimi BLB, Stelfox HT, Turgeon AF, Lauzier F (2015). Impact of socio-economic status on hospital length of stay following injury: a multicenter cohort study. BMC Health Serv Res.

[CR46] Morris TP, White IR, Royston P (2014). Tuning multiple imputation by predictive mean matching and local residual draws. BMC Med Res Methodol.

[CR47] Priest N, Mackean T, Davis E, Briggs L, Waters E (2012). Aboriginal perspectives of child health and wellbeing in an urban setting: developing a conceptual framework. Health Sociol Rev.

[CR48] Priest N, Thompson L, Mackean T, Baker A, Waters E. 'Yarning up with koori kids' - hearing the voices of Australian urban indigenous children about their health and well-being. Ethn Health. 2017;22(6):631–647.

[CR49] Priest NC, Paradies YC, Gunthorpe W, Cairney SJ, Sayers SM (2011). Racism as a determinant of social and emotional wellbeing for Aboriginal Australian youth. Med J Aust.

[CR50] Purdie N, Dudgeon P, Walker R (2012). Working together: Aboriginal and Torres Strait Islander mental health and wellbeing principles and practice.

[CR51] Randall DA, Lujic S, Leyland AH, Jorm LR (2013). Statistical methods to enhance reporting of Aboriginal Australians in routine hospital records using data linkage affect estimates of health disparities. Aust N Z J Public Health.

[CR52] Read DJ, Tan SC, Ward L, McDermott K (2018). Burns first aid treatment in remote northern Australia. Burns..

[CR53] Riedlinger DI, Jennings PA, Edgar DW, Harvey JG, Cleland MHJ, Wood FM (2015). Scald burns in children aged 14 and younger in Australia and New Zealand—an analysis based on the burns registry of Australia and New Zealand (BRANZ). Burns..

[CR54] Russell DJ, Wakerman J, Humphreys JS (2013). What is a reasonable length of employment for health workers in Australian rural and remote primary healthcare services?. Aust Health Rev.

[CR55] Ryder C, Mackean T, Hunter K, Williams H, Clapham K, Holland AJA (2019). Equity in functional and health related quality of life outcomes following injury in children - a systematic review. Crit Public Health.

[CR56] Ryder C, Mackean T, Ullah S, Burton H, Halls H, McDermott D (2017). Development and validation of a questionnaire to measure attitude change in health professionals after completion of an Aboriginal health and cultural safety training Programme. Aust J Indigenous Educ.

[CR57] Salmon M, Skelton F, Thurber KA, Bennetts Kneebone L, Gosling J, Lovett R (2019). Intergenerational and early life influences on the well-being of Australian Aboriginal and Torres Strait islander children: overview and selected findings from footprints in time, the longitudinal study of indigenous children. J Dev Orig Health Dis.

[CR58] Shahid S, Durey A, Bessarab D, Aoun SM, Thompson SC (2013). Identifying barriers and improving communication between cancer service providers and Aboriginal patients and their families: the perspective of service providers. BMC Health Serv Res.

[CR59] Sherwood J (2009). Who is Not Coping with Colonization? Laying Out the Map for Decolonization. Australas Psychiatry.

[CR60] Sherwood J (2013). Colonisation–It’s bad for your health: the context of Aboriginal health. Contemp Nurse.

[CR61] Sherwood J, Edwards T (2006). Decolonisation: a critical step for improving Aboriginal health. Contemp Nurse.

[CR62] Sierra Zúñiga MF, Castro Delgado OE, Merchán-Galvis AM, Caicedo JCC, Calvache JA, Delgado-Noguera M (2016). Factors associated with length of hospital stay in minor and moderate burns at Popayan, Colombia. Analysis of a cohort study. Burns..

[CR63] Taylor SL, Sen S, Greenhalgh DG, Lawless M, Curri T, Palmieri TL (2017). Not all patients meet the 1 day per percent burn rule: a simple method for predicting hospital length of stay in patients with burn. Burns..

[CR64] Tracy L, McInnes J, Gong J, Gabbe B, Thomas T. Burns Registry of Australia and New Zealand Annual Report July 2016–July 2017. Branz: BRANZ, Monash University; 2017. Report No.: 8th Annual Report.

[CR65] Waddell C, Dibley M (1986). The medicalization of Aboriginal children: a comparison of the lengths of hospital-stay of Aboriginal and non-Aboriginal children in Western Australia and the Northern Territory. J Paediatr Child Health.

[CR66] Wakerman J, Humphreys JS, Wells R, Kuipers P, Entwistle P, Jones J (2008). Primary health care delivery models in rural and remote Australia – a systematic review. BMC Health Serv Res.

[CR67] Walker M, Fredericks B, Mills K, Anderson D (2014). “Yarning” as a method for community-based health research with indigenous women: the indigenous women's wellness research program. Health Care Women Int.

[CR68] Walter M (2016). Social exclusion/inclusion for urban Aboriginal and Torres Strait islander people. Soc Inclusion.

[CR69] Walter M (2018). The voice of indigenous data: beyond the markers of disadvantage. Griffith Rev.

[CR70] Walter M, Andersen C (2013). Indigenous statistics: a quantitative research methodology.

[CR71] Walter M, Suina M (2019). Indigenous data, Indigenous methodologies and Indigenous data sovereignty. Int J Soc Res Methodol.

[CR72] Wang T, Nie C, Zhang H, Zeng X, Yu H, Wei Z, et al. Epidemiological characteristics and factors affecting length of hospital stay for children and adults with burns in Zunyi, China: a retrospective study. PeerJ. 2018;6:e5740. 10.7717/peerj.5740 PMC6173946 30310756

[CR73] Weedon M, Potterton J (2011). Socio-economic and clinical factors predictive of paediatric quality of life post burn. Burns..

[CR74] Zubrick SR, Lawrence DM, Silburn SR, Blair E, Milroy H, Wilkes T (2004). The Western Australian Aboriginal child health survey: the health of Aboriginal children and young people.

