# Supplementary material for: Factors contributing to longer length of stay in Aboriginal and Torres Strait Islander children hospitalised for burn injury
Source: Inj Epidemiol. 2020 Oct 5;7:52. doi: 10.1186/s40621-020-00278-7 (PMC7534159; doi:10.1186/s40621-020-00278-7)

Supplementary File 1, Figure A – Knowledge interface methodology, comprising of Indigenous and Western knowledge system for this study


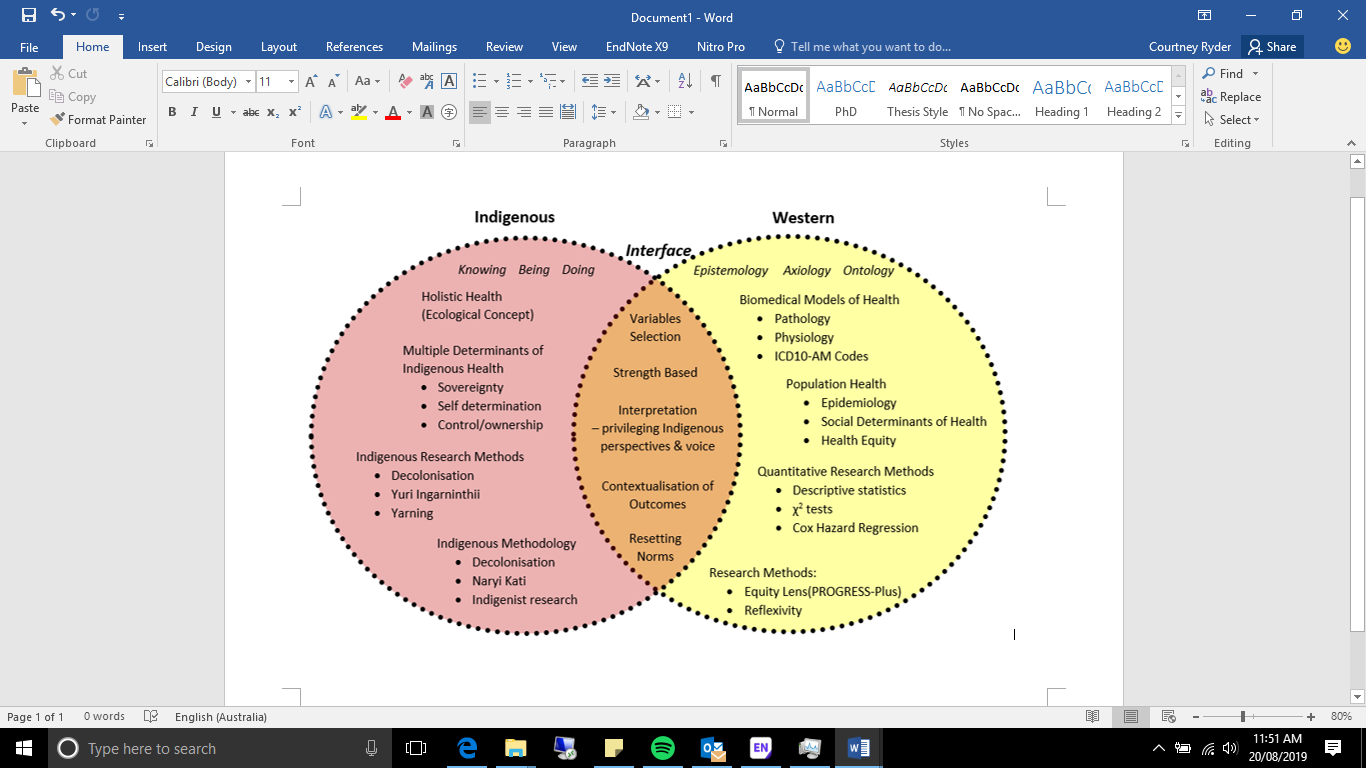

Supplement: Supplementary file 1 — Additional file 1 Fig. A. Knowledge interface methodology, comprising of Indigenous and Western knowledge system for this study. [file 40621_2020_278_MOESM1_ESM.docx]
