# Supplementary material for: Factors contributing to longer length of stay in Aboriginal and Torres Strait Islander children hospitalised for burn injury
Source: Inj Epidemiol. 2020 Oct 5;7:52. doi: 10.1186/s40621-020-00278-7 (PMC7534159; doi:10.1186/s40621-020-00278-7)
Supplement: Supplementary file 2 — Additional file 2 Table B. Variables extracted and created from data source with their definitions. [file 40621_2020_278_MOESM2_ESM.docx]

Supplementary File -2, Table B – Variables extracted and created from data source with their definitions.

| **Variable** | | Source | Definition |
| --- | --- | --- | --- |
|  | *Items (ICD-10-AM or Items)* |  |  |
| **PROGRESS-Plus** | | |  |
|  | *Place of Residence, Race/ethnicity, Gender, Socio-economic status, All Socio-economic position, Age.* | BRANZ | Equity framework, which provides factors which contribute to unequal health status (1, 2). Note - PROGRESS-Plus was used for variables of: Gender, Age Range, ARIA, SES. |
| **Gender** | | | |
|  | *Male, Female, Intersex/indeterminate* | BRANZ | Socio-cultural identity as reported in the BRANZ (3) |
| **Age** | | | |
|  | *Presented as a range* | BRANZ | Age range, which the child falls into, as reported in the BRANZ. |
| **Accessibility/Remoteness Index of Australia (ARIA)** | | |  |
|  | *RA1 Metropolitan, RA2 Inner Regional, RA3 Outer Regional, RA4 Remote, RA5 Very Remote.* | BRANZ  ABS | Accessibility/Remoteness Index of Australia. A geographical classification, based on the population size of a postcode to major centres (4, 5). |
| **Socioeconomic Status, Socio-Economic Indexes for Area (SES)** | | |  |
|  | *1-2 Very Low SES, 3-4 Low SES, 5-6 Medium SES, 7-8 High SES, 9-10 Very High SES* | BRANZ  ABS | Australian Bureau of Statistics measures, which positions residential location postcodes on socio-economic advantage and disadvantage over a 10-point scale (5, 6). |
| **Length of Stay (LOS)** in Hospital | | | |
|  | *Continuous variable* | BRANZ | Day of admission to day of discharge. |
| **Injury Cause** | | | |
|  | *Scald, Contact, Flame, Friction, Other (*electrical, chemical, no cause of other) | BRANZ | Cause of the burns injury as reported in the BRANZ. |
| **Percentage Total Body Surface Area (%TBSA)** (7) | | | |
|  | *T31.00* | BRANZ  ICD-10-AM | Burn < 10 %TBSA |
|  | *T31.10, T31.11* |  | Burn 10 - 19 %TBSA |
|  | *T31.20-T31.99* |  | Burn > 19 %TBSA |
| **Burn Depth** (7, 8) | | |  |
|  | *T20.1, T21.10-.19, T22.10-.12, T23.1, T24.1, T25.1, T26.1, T27.1, T28.1, T29.1, T30.1* | BRANZ  ICD-10-AM | Superficial Thickness |
|  | *T20.2, T21.20-.29, T22.20-.22, T23.2, T24.2, T25.2, T26.2, T27.2. T28.2, T29.2, T30.2* |  | Partial Thickness |
|  | *T20.3, T21.30-.39, T22.30-.32, T23.3, T24.3, T25.3, T26.3, T27.3, T28.3, T29.3, T30.3* |  | Full Thickness |
| **Bacterial Infection** (7) | | | |
|  | *B95.0-B95.5, B95.41, B95.42, B95.48* | BRANZ  ICD-10-AM | Streptococcus Infection (7) |
|  | *B95.6-95.8* |  | Staphylococcus Infection |
| **Burn Dressing** (7) | | | |
|  | *30010-00* | BRANZ  ICD-10-AM | < 10 % Body Surface Area |
|  | *30014-00* |  | ≥ 10% Body Surface Area |
| **Burn Debridement** (7) | | | |
|  | *90686-00* | BRANZ  ICD-10-AM | Nonexcisional |
|  | *30017-01, 30020-00* |  | Excisional |
| **Split Skin Graft** (7) | | | |
|  | *45406-00* | BRANZ  ICD-10-AM | > 3 % Body Surface Area |
|  | *45409-00, 45412-00, 45415-00, 45418-00, 45460-00, 45464-00, 45468-00, 45471-00, 45474-00, 45477-00, 45480-00, 45483-00* |  | ≤ 3 % Body Surface Area  (grafting as ≤ 3% includes 3-6, 6-9, 9-12, 12-15, 15-20, 20-30… > 80%) |
| **Allied Health Intervention** (7) | | | |
|  | *95550-02, -03* | BRANZ  ICD-10-AM | Occupational Therapy or Physiotherapy |
|  | *95550-01, -10* |  | Social Worker or Psychology |
|  | *95550-00, -05, -06, -08, -09* |  | Dietetics, Speech Pathology, Audiology, Prosthetics and Orthotics, or Pharmacy |

**References**

1. Kavanagh J, Oliver S, Lorenc T. Reflections on developing and using PROGRESS-Plus. Equity Update. 2008;2(1):1-3.

2. Ryder C, Mackean T, Hunter K, Williams H, Clapham K, Holland AJA, et al. Equity in functional and health related quality of life outcomes following injury in children - a systematic review. Critical Public Health. 2019:1-15.

3. Gahagan J, Gray K, Whynacht A. Sex and gender matter in health research: addressing health inequities in health research reporting. International Journal for equity in health. 2015;14(1):12.

4. Postcode 2012 to Remoteness Area 2011 [Internet]. Australian Bureau of Statistics. 2011. Available from: <http://www.abs.gov.au/>

5. Australia_Post. Standard Postcode File Australia: Australia Post; 2017 [Available from: <https://postcode.auspost.com.au/>.

6. ABS. Socio-Economic Indexes for Areas (SEIFA). Australia: Australian Bureau of Statistics; 2011. Contract No.: 2033.0.55.001.

7. Australian_Consortium_for_Classification_Development. The international statistical classification of diseases and related health problems, tenth revision, Australian modification (ICD-10-AM/ACHI/ACS). Darlinghurst, NSW: Independent Hospital Pricing Authority; 2017

8. G Shakespeare P. Standards and quality in burn treatment2002. 791-2 p.
