# Supplementary material for: Factors contributing to longer length of stay in Aboriginal and Torres Strait Islander children hospitalised for burn injury
Source: Inj Epidemiol. 2020 Oct 5;7:52. doi: 10.1186/s40621-020-00278-7 (PMC7534159; doi:10.1186/s40621-020-00278-7)
Supplement: Supplementary file 3 — Additional file 3. Table C: Patient characteristics. [file 40621_2020_278_MOESM3_ESM.docx]

| **Supplementary File 3, Table C** | | | | |
| --- | --- | --- | --- | --- |
| **STAGE 1: PATIENT CHARACTERISTICS** | | | | |
|  | **Aboriginal & Torres Strait Islander**  **(n = 723, 10.4%)** | | **Other Australian**  **(n = 6257, 89.6%)** | |
| **Variable** | **Count** | **Percentage (Confidence Intervals)** | **Count** | **Percentage (Confidence Intervals** |
| **Gender*** | | | | |
| Female | 276 | 38.2 (34.7-41.8) | 2443 | 39.0 (37.8-40.3) |
| Male | 447 | 61.8 (58.2-65.3) | 3812 | 60.9 (59.7-62.1) |
| **Age** | | | | |
| < 1 Years | 50 | 6.9 (5.3-9.0) | 744 | 11.9 (11.1-12.7) |
| 1- 4 Years | 384 | 53.1 (49.5-56.7) | 3310 | 52.9 (52.7-54.1) |
| 5 - 9 Years | 154 | 21.3 (18.5-24.4) | 1072 | 17.1 (16.2-18.1) |
| 10 - 15 Years | 135 | 18.7 (16.0-21.7) | 1131 | 18.1 (17.1-19.1) |
| **Accessibility/Remoteness Index of Australia** | | | | |
| Metropolitan (RA1) | 173 | 23.9 (21.0-27.2) | 3819 | 61.0 (59.8-62.2) |
| Inner Regional (RA2) | 108 | 14.9 (12.5-17.7) | 1571 | 25.1 (24.1-26.2) |
| Outer Regional (RA3) | 209 | 28.9 (25.7-32.3) | 677 | 10.8 (10.1-11.6) |
| Remote (RA4) | 121 | 16.7 (14.2-19.6) | 132 | 2.1 (1.8-2.5) |
| Very Remote (RA5) | 113 | 15.6 (13.2-18.5) | 58 | 0.9 (0.7-1.2) |
| **Index of Relative Socio-Economic Advantage and Disadvantage** | | | | |
| Very Low: 1-2 | 370 | 51.2 (47.5-54.8) | 1571 | 25.1 (24.1-26.2) |
| Low: 3-4 | 152 | 21.0 (18.2-24.1) | 1175 | 18.8 (17.8-19.8) |
| Medium: 5-6 | 91 | 12.6 (10.4-15.2) | 1410 | 22.5 (21.5-23.6) |
| High 7-8 | 66 | 9.1 (7.2-11.5) | 975 | 15.6 (14.7-16.5) |
| Very High: 9-10 | 44 | 6.1 (4.6-8.1) | 1125 | 18.0 (17.1-19.0) |
| **STAGE 2: INJURY CAUSE** | | | | |
| Scald | 285 | 39.4 (35.9-43.0) | 3192 | 51.0 (49.8-52.3) |
| Contact | 229 | 31.7 (28.4-35.2) | 1587 | 25.4 (24.3-26.5) |
| Flame | 141 | 19.5 (16.8-22.6) | 662 | 10.6 (9.8-11.4) |
| Friction | 29 | 4.0 (2.8-5.7) | 519 | 8.3 (7.6-9.0) |
| Other** | 39 | 5.4 (4.0-7.3) | 296 | 4.7 (4.2-5.3) |
| **STAGE 3: INJURY SEVERITY** | | | | |
| **Total Body Surface Area %** | | | | |
| Unknown | 26 | 3.6 (2.5-5.2) | 203 | 3.3 (2.8-3.7) |
| < 10% | 612 | 84.7 (81.8-87.1) | 5450 | 87.1 (86.3-87.9) |
| 10-19% | 61 | 8.4 (6.6-10.7) | 461 | 7.4 (6.8-8.0) |
| > 19% | 24 | 3.3 (2.2-4.9) | 147 | 2.4 (2.0-2.9) |
| **Depth of injury** | | | | |
| Superficial thickness | 63 | 8.7 (6.7-11.0) | 695 | 11.1 (10.4-11.9) |
| Partial thickness | 547 | 75.7 (72.4-78.6) | 4839 | 77.3 (76.3-78.4) |
| Full thickness | 113 | 15.6 (13.2-18.5) | 723 | 11.6 (10.8-12.4) |
| **Bacterial Infection** | | | | |
| Streptococcus | 65 | 9.0 (7.1-11.3) | 131 | 2.1 (1.8-2.5) |
| Staphylococcus | 104 | 14.4 (12.0-17.1) | 497 | 7.9 (7.3-8.6) |
| **STAGE 4: TREATMENT** | | | | |
| **Treatment - Dressing** | | | | |
| < 10% Body Surface Area | 83 | 11.5 (9.2-13.8) | 884 | 14.1 (13.3-15.0) |
| ≥ 10% Body Surface Area | 35 | 4.8 (3.3-6.4) | 237 | 3.8 (3.3-4.3) |
| **Treatment – Debridement** | | | | |
| Nonexcisional | 36 | 5.0 (3.4-6.6) | 256 | 4.1 (3.6-4.6) |
| Excisional | 88 | 12.2 (8.9-15.5) | 702 | 11.2 (10.2-12.3) |
| **Treatment - Split Skin Graft** | | | | |
| < 3% Body Surface Area | 61 | 8.4 (6.4-10.5) | 742 | 11.9 (11.1-12.7) |
| > 3%† Body Surface Area | 79 | 10.9 (7.1-14.8) | 455 | 7.3 (6.2-8.3) |
| **Treatment - Allied Health Intervention** | | | | |
| Physical/OT Therapy | 235 | 32.6 (29.2-36.1) | 1355 | 21.7 (20.6-22.7) |
| Social Work & Psychology | 262 | 36.4 (32.9-39.9) | 1614 | 25.8 (24.7-26.9) |
| Other†† Allied Health | 147 | 20.5 (17.5-23.4) | 963 | 15.4 (14.5-16.3) |
| * Gender X - Intersex/indeterminate not included in table, < 0.00%. **Other: electrical, chemical, no cause recorded. † grafting as <3% includes 3-6, 6-9, 9-12, 12-15, 15-20, 20-30…>80%. †† Other: dietetics, speech pathology, audiology, prosthetics, orthotics, pharmacy. OT – Occupational Therapy | | | | |
